# Hearing dogs for people with severe and profound hearing loss: a wait-list design randomised controlled trial investigating their effectiveness and cost-effectiveness

**DOI:** 10.1186/s13063-021-05607-9

**Published:** 2021-10-14

**Authors:** Lucy Stuttard, Philip Boyle, Caroline Fairhurst, Catherine Hewitt, Francesco Longo, Simon Walker, Helen Weatherly, Emese Mayhew, Bryony Beresford

**Affiliations:** 1 Social Policy Research Unit, Department of Social Policy and Social Work, Church Lane Building, York Science Park, University of York, Heslington, YO10 5DF UK; 2 York Trials Unit, University of York, York, YO10 5DD UK; 3 Centre for Health Economics, University of York, York, YO10 5DD UK

## Abstract

**Background:**

Hearing loss increases the risk of poor outcomes across a range of life domains. Where hearing loss is severe or profound, audiological interventions and rehabilitation have limited impact. Hearing dogs offer an alternative, or additional, intervention. They live permanently with recipients, providing sound support and companionship.

**Methods:**

A single-centre, randomised controlled trial (RCT) evaluated the impacts of a hearing dog on mental well-being, anxiety, depression, problems associated with hearing loss (responding to sounds, fearfulness/social isolation), and perceived dependency on others. Participants were applicants to the UK charity ‘Hearing Dogs for Deaf People’. Eligibility criteria were as follows: first-time applicant; applying for a hearing dog (as opposed to other support provided by the charity). Participants were randomised 1:1 to the following: receive a hearing dog sooner than usual [HD], or within the usual application timeframe (wait-list [WL] comparator). The primary outcome was mental well-being (Short Warwick-Edinburgh Mental Well-Being Scale) 6 months (T1) after HD received a hearing dog. The cost-effectiveness analysis took a health and social care perspective.

**Results:**

In total, 165 participants were randomised (HD *n* = 83, WL *n* = 82). A total of 112 (67.9%) were included in the primary analysis (HD *n* = 55, WL *n* = 57). At T1, mental well-being was significantly higher in the HD arm (adjusted mean difference 2.53, 95% CI 1.27 to 3.79, *p* < 0.001). Significant improvements in anxiety, depression, functioning, fearfulness/social isolation, and perceived dependency, favouring the HD arm, were also observed. On average, HD participants had used fewer statutory health and social care resources. In a scenario whereby costs of provision were borne by the public sector, hearing dogs do not appear to be value for money. If the public sector made a partial contribution, it is possible that hearing dogs would be cost-effective from a public sector perspective.

**Conclusions:**

Hearing dogs appear to benefit recipients across a number of life domains, at least in the short term. Within the current funding model (costs entirely borne by the charity), hearing dogs are cost-effective from the public sector perspective. Whilst it would not be cost-effective to fully fund the provision of hearing dogs by the public sector, a partial contribution could be explored.

**Trial registration:**

The trial was retrospectively registered with the International Standard Randomised Controlled Trial Number (ISRCTN) registry on 28.1.2019: ISRCTN36452009.

**Supplementary Information:**

The online version contains supplementary material available at 10.1186/s13063-021-05607-9.

## Background

One in twenty of the world’s population currently live with a hearing loss which impacts their everyday lives. This figure is projected to double by 2050 as the world population ages [1]. Prevalence varies between countries, likely due to differences in age demographics [2]. In the United Kingdom (UK), one in six adults have hearing loss and around one in a hundred are severely or profoundly deaf [3]. Hearing loss, particularly acquired hearing loss in adulthood, is associated with increased risk of poorer quality of life and negative impacts on many life domains including social networks and social inclusion, work and mental and physical health [4–9]. Hearing loss is also associated with cognitive decline and increased risk of dementia [10, 11]. The severity of hearing loss increases the risk for, and severity of, negative impacts on people’s lives.

For those with severe or profound hearing loss, hearing aids offer little benefit [3]. Cochlear implants may be an option for some individuals and are likely to yield greater benefit than hearing aids, particularly in terms of supporting one-to-one communication. However, they can only partially ameliorate the impact of hearing loss on managing and participating in everyday life [12, 13]. Where people are unable to benefit, or achieve limited benefit, from audiological interventions, the focus shifts to adaptation to hearing loss and the prevention (or minimisation) of adverse outcomes [3]. Rehabilitation interventions, addressing speech perception/communication skills, functional adaptation (including equipment provision) and emotional support needs, may be available. However, these are short term and typically provided soon after experiencing a significant deterioration in hearing. Overall, evidence on their effectiveness is poor quality and equivocal, particularly in terms of sustained benefit [14–16].

A further, or alternative, type of rehabilitative intervention is to acquire a hearing dog. Hearing dogs are animals specifically trained to provide ‘sound support’; that is, the ability to respond to common ‘daily living’ sounds (e.g. doorbell, fire alarm, family member calling name) and to use different behaviours to alert and identify the sound to the deaf individual. Similar to other assistance dogs (e.g. guide dogs), there are accredited charities providing hearing dogs in many European countries, North America, Australia and New Zealand. Accredited hearing dog providers adhere to international standards for the assistance dog industry related to dog training and assessment, supporting the ‘hearing dog partnership’, and dog welfare [17]. Whilst ownership is retained by the hearing dog provider, the dog lives permanently with the recipient becoming, as a result, a companion as well as providing sound support [18]. As with other assistance dogs, hearing dogs are (though varying in degree between countries) legally entitled to access public, workplace and commercial/business spaces which typically prohibit pet dogs [19]. Hearing dog recipients are encouraged to take their hearing dog with them whenever they leave their home. On these occasions, the dog wears a jacket that clearly conveys it is a working dog supporting someone with hearing loss. The hearing dog partnership is supported by the provider via on-going, individualised support.

Existing research evidence [20–26] on the impacts of hearing dogs on people’s lives is limited and study designs preclude robust conclusions being drawn. However, they do indicate that hearing dogs may have the potential to affect a range of outcomes. That hearing loss increases the risk for poorer outcomes across a wide range of life domains (and resultant demands on public sector services), and the fact that audiological and rehabilitation interventions appear to have limited impact, make a strong case to investigate the effectiveness and cost-effectiveness of hearing dogs. In terms of economic evaluations, Lundqvist et al. [27] recently undertook a cost-effectiveness analysis of ‘certified service dogs’. However, study participants (*n* = 3) with hearing dogs were not included in the effectiveness study [28].

Internationally, the number of, and demand for, hearing and other assistance dogs is increasing [25, 29]. Currently, such interventions are outwith [21] statutory support with the public via charitable donations (or in some countries, individuals) funding this provision. However, the trend in some countries towards offering individuals the option of holding a budget with which to manage their health/social care arrangements opens up the possibility of the state (indirectly) contributing to such interventions, if only in allowing their use to support the dog’s living costs. Health and social work/care professionals therefore need robust evidence on which to provide information and advice to people with hearing loss. Individuals with hearing loss also need access to high-quality evidence to support decision-making. Having a hearing dog is not an insignificant decision; hearing dog partnerships typically last around 10 years, added to that are the impacts on daily routines and family life that come with having a dog in the household.

This paper reports on a pragmatic, randomised controlled trial which sought to evaluate the impact of a hearing dog on the lives of recipients compared to those not yet in receipt of a hearing dog. The trial included a nested economic evaluation. The setting for the study was Hearing Dogs for Deaf People [30], the only organisation accredited to provide hearing dogs to UK residents [17]. Study objectives were as follows:

- To determine the impact of a hearing dog partnership on mental well-being 6 months post-receipt of a hearing dog, compared with individuals who have not yet received their hearing dog.
- To determine the impact of a hearing dog partnership on secondary outcomes of impairment in anxiety, depression, functioning and hearing loss-associated difficulties 6 months post-receipt of a hearing dog.
- To conduct a nested economic evaluation to investigate the cost-effectiveness of hearing dogs.

The study protocol has been published [31] and this includes details of other elements of the research, including collecting initial, exploratory data on longer-term outcomes and a nested, longitudinal qualitative study. Findings from these will be published elsewhere. As well as being the first RCT to evaluate hearing dogs, it is also only the second randomised trial within the wider field of research on assistance dogs for sensory or motor impairments, the other one being a trial in the 1990s of assistance dogs for people with mobility impairments [32].

## Methods

### Trial design

We conducted a single-centre, superiority, randomised controlled trial (RCT) using a 1-1 allocation ratio and with a nested economic evaluation. Full study details are described in the protocol [31] and a CONSORT checklist is available (Additional file 1). The trial was registered retrospectively (ISRCTN36452009). The protocol omitted in error to state that outcomes data collection included a single item question on perceived dependency on others. There are no other changes to the published protocol. The trial took place between March 2017 and January 2020.

### Intervention

The study concerned hearing dogs bred and trained by Hearing Dogs for Deaf People (HDfDP) (www.hearingdogs.org.uk), the UK’s only accredited hearing dog provider [17].

The intervention was receipt of a hearing dog. The comparator was no hearing dog (using a wait-list design). An overview of HDFDP’s breeding and training programmes, and the process by which a hearing dog is matched to and placed with an applicant to HDfDP are outlined below. Further information can be obtained from HDfDP.

#### HDfDP’s breeding and training programmes

HDfDP only works with the following dog breeds: labrador retriever, cocker spaniel, miniature poodle and mixed breed cockapoos and has its own puppy breeding programme. Brood bitches live with volunteers in regular households [33], and this is where puppies spend their first 8 weeks, with initial socialising work introduced at 5 weeks [34]. At 8 weeks, puppies are placed with a puppy training volunteer in a regular household, living with the volunteer for around 16 months. A four-stage, rewards-based training programme [35] is implemented by the volunteer during this period, with regular supervision and support from HDfDP’s Puppy Training Team. The standards required to progress through the stages of training and be registered as hearing dog adhere to international standards for the assistance dog industry [17, 35]. Dogs are typically placed within a month of passing hearing dog accreditation [36]. The following section describes the application, matching and placement process.

#### The application, matching and placement process

Individuals with severe or profound hearing loss are eligible to apply to HDfDP for a hearing dog. HDfDP aim to pair an applicant with a hearing dog within 2.5–3 years of their application. Medical evidence of severe or profound hearing loss is required for an application to be accepted. A detailed assessment process follows, typically taking 3 to 6 months, during which a profile of the applicant is created. Dogs nearing end of their training (at ~ 20 months old) are screened against these profiles in order to identify potential applicant/hearing dog ‘matches’. A number of factors are taken into account when identifying potential matches. These include any preferences regarding breed and size of dog, applicant and dog temperament, applicant life-style (e.g. sedentary vs active) and household (e.g. presences of children or cats), location and the settings where the dog will work (e.g. nature of workplace, use of public transport).

Once an applicant is matched with a specific dog, the match is assessed over a 2-day, residential period. If successful, the dog undergoes further training specific to the settings in which they will be working (e.g. work or travelling environment, idiosyncratic sounds) before permanent placement with the recipient. In the early days, the placement is closely monitored and supported on a needs basis by a HDfDP partnership instructor (PIs) via home visits. During the first 12 months, this gradually decreases in intensity. After that, PIs visit at least annually and can be contacted at any time by the hearing dog recipient.

#### HDfDP need categorisation

The HDfDP assessment includes assigning an applicant to one of four categories of need pertinent to matching them with a hearing dog:

- No remarkable/particular needs,
- Predominantly personal needs (e.g. particular health concerns, mobility issues),
- Predominantly environmental needs (e.g. inner city location, cats in the home),
- Both personal and environmental needs.

### Study participants

Participants were applicants to HDfDP. HDfDP opens for applications four times a year (on-line or postal). During each round, they receive in excess of 300 applications, of which around fifty are offered the opportunity to progress their application (dependent on projected volume of dogs nearing the end of training). HDfDP’s criteria for accepting an application are as follows: individual has severe or profound hearing loss (evidenced by audiology report); is able to meet the welfare, physical and training needs associated with having a hearing dog (evidenced by a report from the applicant’s general practitioner (GP)); and any dog in the household is 10 years or older.

Trial inclusion criteria:

- Application for a hearing dog accepted by HDfDP
- First-time applicant to HDfDP
- Applying for a hearing dog
- Aged 18 or over.

Trial exclusion criteria:

- Applying for a dual assistance (sound and vision) or companion dog
- Applying for a replacement hearing dog due to impending retirement or death of/previous hearing dog
- Presence of cognitive impairment (indicated by use of proxy during application process).

### Recruitment and baseline data collection

HDfDP screened all accepted applications between March 2017 and March 2018 against study eligibility criteria and posted a Study Recruitment Pack to those fulfilling the criteria. Recruitment materials clearly stated the research team was independent of HDfDP and no data would be shared with the organisation. Individuals deciding to join the study returned the consent form and baseline questionnaire to the research team at the University of York. Print versions of recruitment materials included details of how to access electronic versions (English or British Sign Language [BSL]) hosted on the Qualtrics© survey 7 (platform). Participants received a £20 shopping voucher as a ‘thank you’ for returning their baseline questionnaire.

### Randomisation

As soon as the profiles of two participants with the same HDfDP need categorisation were completed, pairwise randomisation was used to allocate one each to receive a hearing dog:

- Sooner than usual (hearing dog (HD) arm); or
- Within HDfDPs usual timeframe (wait-list (WL) comparator arm)

Participants were blinded to their group allocation.

Randomisation was carried out by a trial statistician at York Trials Unit using an allocation schedule generated in Stata v15. Study team members who were actively involved in the administration of the trial were not blinded.

After randomisation, HD profiles were immediately made available to HDfDP trainers to identity a suitable hearing dog ‘match’. WL profiles were not made available until 6 months after HD received their dog or, to fulfil HDfDP’s commitment to receive a hearing dog within 2.5 to 3 years of applying, 16 months after randomisation, whichever came first.

The study adhered to HDfDP protocols regarding cessation of a hearing dog partnership. Cessation may be instigated by the recipient (e.g. significant deterioration in their health), the organisation (e.g. concerns about dog welfare) or where recipient and the organisation jointly decide that the match has not proved successful (e.g. dog behaviour issues, mis-match in dog and recipient vitality).

Pre-randomisation, participants who withdrew their application for a hearing dog or had their application closed by HDfDP (due to evidence emerging during the assessment process that the participant did not meet HDfDP acceptance criteria) were no longer eligible for participation in the trial. Post-randomisation, all participants, regardless of their application or partnership status remained within the trial unless they withdrew consent. Where a HD participant withdrew their application post-randomisation, the research team created a dummy partnership date from which to trigger T1 data collection. If HDfDP closed an application post-randomisation or removed the hearing dog, they had the option to request that the participant was removed from the study.

### Follow-up data collection

The first follow-up data collection (T1) for each randomised pair took place 6 months after the HD participant received a hearing dog, at which point the WL counterpart was not expected to have received their dog. This was the primary time point. The decision to use 6 months after receipt of hearing dog as the primary time point was informed by two main considerations: (i) advice from HDfDP that, by that time, the settling in and adjustment period has typically passed and the partnership between individual and hearing dog well-established; (ii) ensuring HDfDP would maintain its commitment that all applicants (including those in the WL group) receive a hearing dog within 2.5–3 years of their application.

Follow-up data collection was administered by the research team via post or email (containing link to electronic versions (English or BSL) of study questionnaire) according to participants’ preferences. Email/post and text reminders, and ‘thank you’ shopping vouchers (£20) supported retention.

### Outcome measures

The selection of outcome measures was informed by the existing literature and in consultation with Hearing Dogs for Deaf People and hearing dog recipients.

#### Primary outcome

Self-reported mental well-being was measured using the 7-item *Short Warwick-Edinburgh Mental Well-being Scale (SWEMWBS*) [37]. This is scored between 7 and 35 and can be classified to indicate low (7–19.3), medium (19.4–28.0) and high (28.1–35) well-being [38]. Internal reliability for the study sample was excellent *a* = 0.90.

#### Secondary outcomes

*Work and Social Adjustment Scale (WSAS)* [39] measures perceived functional impairment associated with a specified health problem/disability with respect to five domains (work, home management, social leisure activities, private leisure activities and relationships with others) each represented by a single item. It yields a score between 0 and 40 (0–9 indicates low impairment, 10–19 moderate impairment, and 20–40 severe impairment). Internal reliability for the study sample was good *a* = 0.84.

*Generalised Anxiety Disorder Assessment* (GAD-7) [40] is a seven-item instrument measuring anxiety. A 4-point rating scale (0–3) for each item yields a total score between 0 and 21 (0–4 indicates minimal anxiety, 5–9 mild, 10–14 moderate, and 15–21 severe). Internal reliability for the study sample was excellent *a* = 0.93.

*Patient Health Questionnaire* (PHQ-9) [41] is a nine-item instrument measuring depression. A 4-point rating scale (0–3) for each item yields a total score between 0 and 27 (0–4 indicates no depression, 5–9 mild, 10–14 moderate, 15–19 moderately severe, and 20–27 severe). Internal reliability for the study sample was excellent *a* = 0.91.

*Hearing Loss Questionnaire (HLQ*) comprises two sub-scales (*HLQ-Sound* (6 items), *HLQ-Social* (5 items)) which capture frequency of exposure to problems deaf people report experiencing in everyday life using a five-point rating scale (1 = never to 5 = almost always). *HLQ-Sound* is scored 6–30 and concerns problems with sound detection/identification. *HLQ-Social* is scored 5–25 and concerns problems with feeling safe, dependence on others and social avoidance. The HLQ was derived from a checklist developed by a previous hearing dog evaluation [22] and which analysed items individually. To determine whether we could treat it as a scale, we analysed baseline data using a classical test theory approach [42]. Tests of face validity and exploratory factor analysis indicated a two-factor structure capturing (i) problems with responding to environmental sounds and dependency on others to act as ‘ears’ (HLQ-Sound), and (ii) fearfulness and social isolation (HLQ-Social). Tests of reliability (alpha co-efficients: HLQ-Sound *a* = .82, HLQ-Social *a* = .85) indicated it was appropriate to use subscale scores. Split-half and test-retest reliability were also acceptable. (Further information available from authors).

*Perceived dependency* is a single question assessed perceived dependency on others: In a typical week, on how many days do you need family or friends to help you overcome any difficulties caused by hearing loss?

Validated BSL versions of the SWEMWBS, GAD-7, PHQ-9 and WSAS were already available [43, 44]. The research team created a BSL version of the HLQ and perceived dependency question.

### Compliance

HDfDP retained the right to allocate a hearing dog to a WL participant before T1 should the applicant’s unusual/complex needs significantly reduce the number of hearing dogs with the required specific aptitudes/skills likely to become available within the 3 years HDfDP commits to applicants receiving a hearing dog. Instances were recorded by the study team.

### Sample size

Recruitment was scheduled for a 12-month period during which we estimated that 200 individuals would be approached to take part and 180 would be eligible for recruitment to the study. We assumed that approximately 90% would opt to join the trial (*n* = 162) and at least 128 (80% of these) would be retained at T1. This would result in 80% power to detect an effect size of 0.5 of a standard deviation in our primary outcome measure (two-sided 5% alpha).

### Statistical analysis

Full details of the statistical analysis plan are available (ISRCTN36452009). Data were entered into SPSS Version 26 by staff not involved in data analysis. A random sample of 10% was double entered to assess data quality. A 5% threshold for the error rate was set to trigger further investigation but the error rate did not exceed this. Management of missing data followed guidance from scale developers where available. For the HLQ, we replaced up to one missing item in each subscale with the subscale mean.

Baseline data were summarised descriptively by trial arm as randomised and as included in the primary analysis. Analysis was conducted using two-sided statistical tests at the 5% significance level using available case intention-to-treat.

The primary analysis estimated the difference in SWEMWBS scores at T1 between HD and WL using linear regression adjusting for the baseline score and the individual’s HDfDP need categorisation. Secondary outcomes (WSAS, GAD-7, PHQ-9, HLQ, Perceived dependency) were analysed in the same way.

Standardised effect sizes were calculated by dividing the adjusted mean difference by the standard deviation of the analysed sample at baseline. An effect size of 0.2 was described as a small effect size, 0.5 a medium effect size and > 0.8 a large effect size [45].

Secondary analyses included a series of ordinal logistic regressions, adjusting for HDfDP needs categorisation and baseline scores, which compared differences between arms at T1 in distribution across severity classifications of the SWEMWBS, GAD7, PHQ-9 and WSAS.

A Complier Average Causal Effect (CACE) analysis [46] was conducted for the primary outcome to estimate the effect of receiving a hearing dog accounting for noncompliance and contamination. A two-stage least squares, instrumental variable (IV) approach was used, adjusting for presenting needs and baseline value of the primary outcome, with randomised group as the independent variable.

### Cost-effectiveness

#### Data collection

The following was collected at baseline, randomisation and T1:

- *Health-related quality of life,* measured using the EuroQol 5 Dimensions (EQ-5D-5L [47]. This profiles health-related quality of life (HRQoL) on the dimensions of mobility, self-care, usual activities, pain/discomfort and anxiety/depression and according to 5 levels of severity (no problems to extreme problems). For the UK, the crosswalk range of scores is − 0.594 to 1 [48].
- A questionnaire developed specifically for the study, and reflecting the specific needs of the population, collected data on *use of primary/community and secondary health care and social care services* in the previous 3 months. (Questionnaire available from the research team)

#### Analytical perspective

The cost-effectiveness analysis took a health and social care perspective which asked, compared to usual care, does providing hearing dogs provide value for money for the National Health Service (NHS) and local authorities (LA)? Or, in other words, does investing public sector resources in hearing dogs generate more benefit than could be generated by alternative spending? The main analysis considered two separate scenarios:

1. The costs of providing hearing dogs is borne by the charity (the current situation)
2. The public sector funds the cost of providing hearing dogs.

Based on findings from the main analysis, a subsequent analysis considered how much the public sector could contribute towards the cost of hearing dogs with the expenditure representing value for money.

#### Data analysis

Health and social care services used by study participants were costed (see Additional file 2 for unit costs; where possible these were based on national average costs). The cost of providing hearing dogs (which includes breeding, puppy training, hearing dog training, assessment and matching applicant to dog, and Partnership Instructor support ) was calculated using HDfDP’s 2017/18 financial accounts. More detail on costs cannot be reported in this paper due to commercial sensitivity. However, these can be made available to research teams on request.

EQ-5D-5L responses were converted onto the EQ-5D-3L instrument using the crosswalk algorithm developed by Van Hout et al. [49] to estimate HRQoL scores as recommended by NICE [50]. Then, health quality-adjusted life-years (H-QALYs) were calculated using the area under the curve method [51]. H-QALYs are a generic measure of health which captures both quantity and quality of life with one H-QALY representing a year spent in perfect health. In addition, the HRQoL scores were converted into the Adult Social Care Outcome Toolkit (ASCOT) score using the exchange rate proposed by Stevens et al. [52] ASCOT scores were used to estimate social care-QALYs (SC-QALYs) using the area under the curve method [51].

For each scenario, we conducted two analyses:

i. The *whole trial period* (baseline to T1)
ii. The active intervention period (receipt of hearing dog to T1)

Table 1 provides more detail about these approaches.

**Table 1:** Description of the whole trial period and the active intervention period: cost-effectiveness analysis

	Whole trial period	Active intervention period
Rationale	To test whether, before receipt of a HD, the idea of receiving a HD might have an impact on differential QALYs and costs.	To capture the potential differential QALYs and costs on receipt of a HD.
Reference period	From baseline to T1. This was calculated as 21 months long. It is the average duration between baseline and T1 minus 6 months when the HD arm received a HD (i.e. approximately 15 months), plus 6 months of the active intervention.	From T1 minus 6 months when the HD arm received the HD, to T1 at 6 months.
Assumptions	QALYs: • HRQoL remains the same from baseline to receipt of the HD by the HD arm. • HRQoL changes linearly from receipt of the HD in the HD arm to T1. Costs: • Resource use in the 3 months prior to baseline is representative of the resource use between baseline and the time in the HD arm to receipt of a HD. • Resource use in the 3 months prior to T1 is representative of the entire 6-month active intervention period.	QALYs: • HRQoL remains the same from randomisation to the HD arm receiving the HD. • HRQoL changes linearly from the HD arm receiving the HD to T1. Costs: • Resource use in the 3 months prior to T1 is representative of the 6-month active intervention period.
Limitations	The calculation of QALYs and costs requires additional assumptions which might not hold true in reality.	This analysis may not capture the differential QALYs and costs potentially incurred had the HD arm been in receipt of a HD at baseline.

Model selection was carried out using Akaike and Bayesian information criterion (AIC and BIC). In the whole trial period analysis, the difference in QALYs and costs between the two arms of the trial were estimated by ordinary least square (OLS). In the active intervention period analysis, the difference in QALYs was estimated using generalised linear model (GLM) with the log-link and normal distribution. The difference in costs in the scenario *excluding* the cost of the hearing dog was estimated using GLM with log-link and gamma distribution. The difference in costs in the scenario *including* the cost of the hearing dog was estimated using OLS. QALY and cost regressions included the EQ-5D-5L score and health and social care costs in natural units at baseline, respectively, in order to adjust for baseline differences across participants. All regressions included three dummies indicating the HDfPD’s ‘needs categorisation’ (the reference category: no remarkable/particular needs). For each time point, missing data were imputed using multiple imputation chain equation with predictive mean matching [53].

Cost-effectiveness was calculated using the incremental cost-effectiveness ratio (ICER), which reports the incremental cost per QALY gained of one intervention compared to another. The incremental net health benefit (NHB) for hearing dogs compared to standard care was also estimated which captures the overall health gain from one individual receiving a hearing dog. This measure reflects any health benefits to that individual, and the impacts on other people’s health from any change in resource requirements. If hearing dogs are cost saving, those resources (or funds) can be used to generate additional health for other individuals, resulting in further health gain. In contrast, if they increase costs those resources are not available for others, resulting in health losses. Incremental net health benefit was estimated using three different cost-effectiveness thresholds: £15,000 [54], £20,000 and £30,000 per QALY [50]. Each of the thresholds represents an estimate of the health opportunity cost (i.e. the QALYs that could have been generated elsewhere from the same resources). To estimate how much the NHS could contribute towards hearing dogs, the maximum price was estimated at which the incremental NHB was still positive, that is how much could be spent on hearing dogs before any health gain generated from hearing dogs was exceeded by health loss elsewhere. The analysis was carried out in Stata 16.

## Results

### Participant flow

**Fig. 1:**
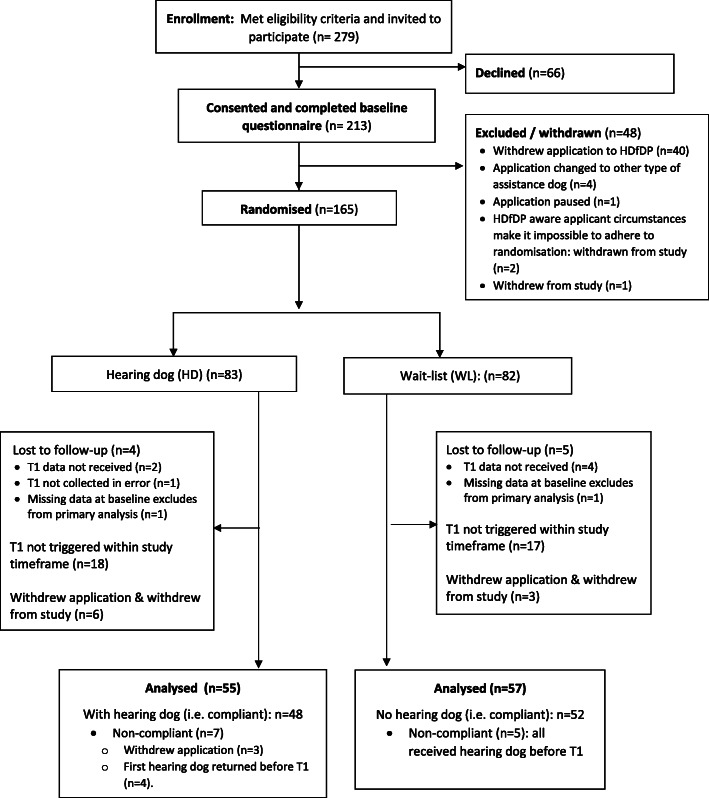
CONSORT flow diagram: enrolment to T1

Figure 1 summarises the flow of participants through the study. In total, 213 individuals were recruited to the study of whom 165 (77%) were randomised (Fig. 1) (HD *n* = 83, 50%; WL *n* = 82, 50%). A total of 112 randomised participants (68%) were included in the primary analysis; 51 (HD *n* = 24; WL *n* = 27) were not followed up at T1 as this was not triggered within the study timeframe (dictated by funding envelope), and two did not provide valid SWEMWBS at baseline. Of those followed up at T1 (and their data included in the analysis), 87% of HD arm participants had received a hearing dog and 91% of WL arm participants were still waiting to receive a hearing dog. Where T1 data collection was attempted, the response rate was 93%.

### Randomised sample: characteristics

Three quarters of the sample were female (*n* = 123) and the average age was 48 years (range 18–86, Table 2). Just under half (49%) were married or living as married, 44% were single or divorced/separated, and 7% widowed. Sixty percent reported having an additional long-term condition (e.g. mental health, mobility, stamina/fatigue).

**Table 2:** Baseline characteristics

	As randomised			Included in primary analysis (trial)		
	Hearing dog (***n*** = 83)	Wait-list (***n*** = 82)	Total (***n*** = 165)	Hearing dog (***n*** = 55)	Wait-list (***n*** = 57)	Total (***n*** = 112)
**Sex** ***n*** **(%)**						
Female	63 (75.9)	60 (73.2)	123 (74.5)	42 (76.4)	41 (71.9)	83 (73.1)
Male	20 (24.1)	22 (26.8)	42 (25.5)	13 (23.6)	16 (28.1)	29 (25.9)
**Age (years)**						
Mean (SD)	47.7 (18.6)	49.7 (17.0)	48.7 (17.8)	48.2 (18.6)	50.6 (17.2)	49.4 (17.8)
Median (Min, max)	47 (18, 86)	50.5 (18, 81)	49 (18, 86)	49 (19, 86)	50 (18, 79)	49 (18, 86)
**Ethnicity** ***n*** **(%)**						
White British	78 (94.0)	80 (97.6)	158 (95.7)	52 (94.5)	56 (98.2)	108 (96.4)
All other	5 (6.0)	2 (2.4)	4 (4.2)	3 (5.5)	1 (1.8)	4 (3.6)
**Relationship status** ***n*** **(%)**						
Single	31 (37.3)	27 (32.9)	58 (35.2)	22 (40.0)	18 (31.6)	40 (35.7)
Married/cohabiting	39 (47.0)	39 (47.6)	78 (47.3)	27 (49.1)	28 (49.1)	55 (49.1)
Separated/divorced	10 (12.0)	11 (13.4)	21 (12.7)	3 (5.5)	6 (10.5)	9 (8.0)
Widowed	3 (3.6)	5 (6.1)	8 (4.8)	3 (5.5)	5 (8.8)	8 (7.1)
**Long-term health conditions (expected to last more than 12 months)** ***n*** **(%)**						
Yes	48 (57.8)	49 (59.8)	97 (58.8)	29 (52.7)	32 (56.1)	61 (54.5)
No	35 (42.2)	33 (40.2)	68 (41.2)	26 (47.3)	25 (43.9)	51 (45.5)
**Prior pet dog experience** ***n*** **(%)**						
Yes	56 (67.5)	54 (65.9)	110 (66.7)	37 (67.3)	39 (68.4)	76 (67.9)
No	27 (32.5)	28 (34.1)	55 (33.3)	18 (32.7)	18 (31.6)	36 (32.1)
**Years with severe/profound hearing loss** ***n*** **(%)**						
Mean (SD)	28.6 (18.1)	24.3^a^ (19.3)	26.5 (18.8)	28.0 (16.5)	23.4^b^ (19.2)	25.68 (18.0)
Median (range)	25 (1–76)	20 (2–76)	22 (1–76)	25 (1–63)	20 (2–76)	22 (1–76)
**HDfDP needs categorisation**^**c**^ ***n*** **(%)**						
None	43 (51.8)	44 (53.7)	82 (52.7)	28 (50.9)	31 (54.4)	59 (52.7)
Personal	12 (14.5)	13 (15.9)	25 (15.2)	6 (10.9)	5 (8.8)	11 (9.8)
Environmental	21 (25.3)	18 (22.0)	39 (23.6)	17 (30.9)	17 (29.8)	34 (30.4)
Both	7 (8.4)	7 (8.5)	14 (8.5)	4 (7.3)	4 (7.0)	8 (7.1)

^a^Missing data for 4 participants

^b^Missing data for 2 participants

^c^ *None* no remarkable/particular needs, *personal* predominantly personal needs (e.g. particular health concerns, mobility issues), *environmental* predominantly environmental needs (e.g. inner city location, cats in the home), *both* personal and environmental needs

Duration of severe/profound hearing loss ranged from 1 to 76 years. In terms of HDfDP’s additional needs classification, 53% had no additional needs, 24% had predominantly environmental needs, 15% had predominantly personal needs, and 8% both personal and environmental needs.

Baseline characteristics were balanced between the two arms as randomised and as analysed (see Table 2). Comparison of the randomised sample with the study population (aggregate data provided by HDfDP, data available on request) indicated the study sample was representative of current applicants.

### Primary analysis

There was evidence of a difference in SWEMWBS score at T1 (Table 3) favouring HD (adjusted mean difference between arms 2.53, 95% CI 1.27 to 3.79, *p* < 0.001; effect size 0.6).

**Table 3:** Linear regression analyses: primary and secondary outcomes

	Hearing dog		Wait-list		Adjusted mean difference (95% CI) ***p*** value	Standardised effect size
	Baseline	T1	Baseline	T1		
**Short Warwick-Edinburgh Mental Well-being Scale**						
n	55		57		2.53 (1.27 to 3.79) *p* < 0.001	0.60
Mean (SD)	21.3 (3.97)	23.0 (4.46)	22.0 (4.25)	20.9 (3.28)		
**Work and Social Adjustment Scale**						
n	55		58		− 3.31 (− 5.84 to − 0.78) *p* = 0.01	0.37
Mean (SD)	17.9 (8.41)	15.6 (7.82)	20.7 (9.23)	20.8 (9.64)		
**Generalised Anxiety Disorder-7 Questionnaire**						
n	55		56		− 2.96 (− 4.44 to − 1.49) *p* < 0.001	0.51
Mean (SD)	6.5 (5.90)	5.1 (4.79)	6.4 (5.66)	8.0 (6.06)		
**Patient Health Questionnaire-9**						
n	56		57		− 2.56 (− 4.20 to − 0.93) *p* = 0.002	0.40
Mean (SD)	7.2 (6.29)	5.8 (5.58)	6.4 (6.47)	7.9 (6.22)		
**Hearing Loss Questionnaire-Sound Subscale**						
n	53		57		− 1.31 (− 0.35 to 2.97) *p* = 0.12	0.31
Mean (SD)	25.0 (4.29)	23.2 (5.84)	24.8 (4.14)	24.4 (4.86)		
**Hearing Loss Questionnaire-Social Subscale**						
n	56		58		− 2.29 (− 3.47 to − 1.12) p < 0.001	0.45
Mean (SD)	14.8 (5.21)	13.7 (4.62)	16.2 (4.94)	16.9 (4.58)		
**Perceived dependency**						
n	49		55		− 1.66 (− 2.51 to 0.82) p < 0.001	0.64
Mean (SD)	5.0 (2.61)	3.5 (2.59)	4.6 (2.61)	5.00 (2.47)		

### Secondary outcomes

With respect to the secondary outcome measures, with the exception of the HLQ-Sound, there was evidence of a difference between the two groups at T1 favouring HD (Table 3). Effect sizes were small (WSAS, PHQ-9, HLQ-Sound, HLQ-Social) or medium (GAD-7, Perceived dependency).

### Further analyses

At T1, categorisation according to SWEMWBS clinical severity classifications differed between HD and WL (see Table 4). For HD, the odds of being categorised as high well-being (versus medium/low well-being) were 4.20 (95% CI 1.76 to 10.05, *p* < 0.001) times those of WL. Similar patterns were observed for the GAD-7 and PHQ-9, but not the WSAS (Table 4).

**Table 4:** Ordinal regression analyses: clinical severity classifications for the SWEMWBS, WSAS, GAD-7 and PHQ9

	***n***	Hearing dog					***n***	Wait-list					Odds ratio^**a**^ (95% CI) ***p*** value
**Short Warwick-Edinburgh Mental Well-being Scale**													
*Severity*		*Low*	*Medium*		*High*			*Low*		*Medium*		*High*	
Baseline	55	23 (41.8)	28 (50.9)		4 (8.8)		57	18 (31.6)		34 (59.6)		5 (8.8)	4.20 (1.76 to 10.05) *p* < 0.001
T1	55	11 (20.0)	37 (67.3)		7 (12.7)		57	21 (36.8)		36 (63.2)		0 (0.0)	
**Work and Social Adjustment Scale**													
*Functional impairment*		*Sub-clinical*	*Less severe*		*Moderately severe or worse*			*Sub-clinical*		*Less severe*	*Moderately severe or worse*		
Baseline	55	11(20.0)	24 (43.6)		20 (36.4)		58	5 (8.6)		25 (43.1)	28 (48.3)		0.55 (0.25 to 1.20) *p* = 0.133
T1	55	13 (23.6)	26 (47.3)		16 (29.1)		58	8 (13.8)		23 (39.7)	27 (46.6)		
**Generalised Anxiety Disorder-7 Questionnaire**													
*Severity classification*		*Below cut-off*	*Mild*		*Moderate*	*Severe*		*Below cut-off*		*Mild*	*Moderate*	*Severe*	
Baseline	55	25 (45.5)	14 (25.5)		10 (18.2)	6 (10.9)	56	24 (42.9)		20 (35.7)	4 (7.1)	8 (14.3)	0.33 (0.15 to 0.71) *p* = 0.005
T1	55	30 (54.5)	17 (30.9)		4 (7.3)	4 (7.3)	56	21 (37.5)		16 (28.6)	10 (17.9)	9 (16.1)	
**Patient Health Questionnaire-9**													
*Severity classification*		*Below cut-off*	*Mild*	*Moderate*	*Moderately severe*	*Severe*		*Below cut-off*	*Mild*	*Moderate*	*Moderately severe*	*Severe*	
Baseline	56	23 (41.1)	14 (25.0)	12 (21.4)	5 (8.9)	2 (3.6)	57	30 (52.6)	14 (24.6)	4 (7.0)	6 (10.5)	3 (5.3)	0.30 (0.14 to 0.65) *p* = 0.002
T1	56	31 (55.4)	13 (23.2)	7 (12.5)	3 (5.4)	2 (3.6)	57	20 (35.1)	16 (28.1)	13 (22.8)	5 (8.8)	3 (5.3)	

^a^ This is a proportional odds ratio, thus, for each and every response category *c*, compares the people who are in categories greater than *c* with those in categories less than or equal to *c*. That is, for a one unit change in the predictor variable, the odds for cases in a category that is greater than *c* versus less than or equal to *c* are *k* times larger, where *k* is the odds ratio

### Complier Average Causal Effect (CACE) analysis

Within the analysed sample, 87% (48/55) of HD arm participants had a hearing dog and 91% (52/57) of WL arm participants had not received a hearing dog at T1 (Fig. 1). The CACE estimate of the effect of receiving a hearing dog was a difference in SWEMWBS score favouring the HD arm of 3.19 (95% CI 1.65 to 4.73, *p* < 0.001, effect size 0.78).

### Cost-effectiveness analysis

As for the effectiveness analysis, the analysed sample (*n* = 165) comprised *n* = 83 (HD arm) and 82 (WL arm). Table 5 shows descriptive statistics and results for the cost-effectiveness analysis for the whole trial period and the active intervention period. Additional file 3 presents, for each time point, descriptive information on health (Table 6) and social care resource use (Table 7), EQ-5D-5L index score, health and social care costs (Table 8), and missing data (Table 9).

**Table 5:** Cost-effectiveness analysis: descriptive statistics and results

Costing scenario^a^	Trial arm				δ^QALY^		δ^COST^		ICER	Net health benefit (NHB)		
	Hearing dog		Wait-list							*λ* = £15,000 per QALY	*λ* = £20,000 per QALY	*λ* = £30,000 per QALY
	QALYs	Costs (£)	QALYs	Costs (£)	Obs	Coeff	Obs	Coeff				
*Multiple imputation analysis for the whole trial period*												
Excluded	1.300	3909	1.256	4407	165	0.012	165	− 260	Dominant	0.029	0.025	0.021
Included		7123						2954***	242,912	− 0.185	− 0.136	− 0.086
*Multiple imputation analysis for the active intervention period*												
Excluded	0.372	911	0.353	1206	165	0.014	165	− 291	Dominant	0.034	0.029	0.024
Included		4125						2,954***	203,959	− 0.182	− 0.133	− 0.084

*Key: δ* adjusted mean difference, *λ* opportunity cost threshold, *Obs* number of observations used to estimate *δ*, *Coeff* estimated coefficient 1, *ICER* incremental cost-effectiveness ratio

^a^ Refers to two scenarios used for each analysis: costs of providing a hearing dog *excluded* (i.e. borne by HDfDP charity: the current situation), or *included* in costs to public sector (health and social care)

*** *p* value = 0.01

For the whole trial period, on average, QALYs for the HD arm were higher than for the WL arm (1.300 vs 1.256). The estimated differential QALY (δ^QALY^) was 0.012 (ns). In the scenario where the cost of a hearing dog is borne entirely by the charity, the estimated differential cost (δ^COST^) was − £260, indicating that public sector care costs were lower for the HD arm. This difference was not statistically significant but, on average, it was driven by 0.6 fewer other outpatient visits (statistically significant at the 1% level) and 0.2 fewer day cases (statistically significant at the 10% level) in the HD arm. When the cost of the hearing dog (£3,214 for the 6-month active intervention period) was included, δ^COST^ was £2954 and was statistically significant, that is total costs were, on average, higher in the HD arm. Discounting QALYs and costs in the second year (from month 13 to 21) at 3.5%, in line with NICE guidance [55], had no impact on the results.

Results for the active intervention period were similar with a marginally higher estimated differential QALY (δ^QALY^) of 0.014, and marginally higher cost savings (δ^COST^) of £291, indicating that public sector care costs were lower for the HD arm. When the cost of the hearing dog was included, δ^COST^ was £2954 and was statistically significant. That is, total costs were, on average, higher in the HD arm.

In the scenario where the cost of the hearing dogs are fully borne by the charity, hearing dogs improved outcomes and reduced costs and were therefore the dominant, cost-effective strategy. In line with this finding, NHB was positive for the whole trial and active intervention period for all three cost-effectiveness thresholds (i.e. £15,000, £20,000 and £30,000 per QALY). The probability that the hearing dog was cost-effective was over 94% for all health opportunity cost thresholds considered.

In contrast, in the scenario where the cost of a hearing dog is fully borne by the public sector, the ICER was £242,912 per QALY for the whole trial analysis period and £203,959 per QALY for the active intervention period. These values are considerably higher than the highest threshold of £30,000 per QALY and indicate that under this scenario, hearing dogs are not cost-effective. This is confirmed by a probability of cost-effectiveness equal to 0% and a negative NHB for all three thresholds. Figure 4 (Additional file 4) provides graphical representation of these findings using the cost-effectiveness plane and the cost-effectiveness acceptability curve for the two scenarios.

When the whole trial period is analysed, the maximum amount the public sector could contribute towards the cost of a hearing dog during this period, and remain value for money, is £442, £503 and £625 at each threshold value respectively. When the active intervention period is analysed, these figures are £509, £581 and £726. Finally, when the analysis is repeated using SC-QALYs, findings are similar (see Table 10, Additional file 5; Figure 3, Additional file 6) and conclusions are unchanged.

## Discussion

This is the first RCT to evaluate the impact of a hearing dog and their cost-effectiveness. Over two thirds of those invited to participate were recruited and the randomised sample represented the target population. Compliance to the protocol was good. It represents a significant step forward in evaluation research on hearing dogs, and within the wider field of assistance dogs where significant methodological weaknesses in existing studies has constrained our understanding of the impacts of assistance dogs, including hearing dogs, on people’s lives [26].

Adults with hearing loss, particularly those who acquire hearing loss in adulthood, are at risk of a number of adverse outcomes including social isolation, mental health difficulties, unemployment, dependence, increased risk of accidents, poorer physical health and impaired quality of life [4–9]. Trial findings indicate that a hearing dog significantly improves recipients’ mental well-being when assessed 6 months post-receipt of a hearing dog. In addition, receiving a hearing dog significantly improved daily functioning, anxiety, depression, fearfulness/social isolation and perceived dependency on others associated with hearing loss. Effect sizes were small or medium suggesting at least some hearing dog recipients had experienced meaningful change in their lives on the domains captured. Findings from the CACE analysis (SWEMWBS only) indicate these may be conservative estimates of effect. The only domain where a statistically significant difference was not found was in difficulties responding to environmental sounds; however, there was a small effect size favouring HD. Finally, at baseline, over one third of participants reported poor mental well-being. At T1, HD participants were four times more likely to be categorised as having high mental well-being compared to WL participants.

These findings align with earlier studies (all with one or more significant study design limitations) which have evaluated the impact of hearing dogs. Guest’s before and after study [22] also reported improvements in scores on standardised measures of mental health and mental well-being [22]. Previous studies have also reported fewer difficulties responding to sounds [22], social isolation [22, 24], and fearfulness [22, 24] and a reduced sense of dependency [24, 25]. In contrast to previous research [21, 25]; however, we also observed significant improvements in quality of life. Our findings align with the wider literature on reporting the wide-ranging impacts of assistance dogs on the lives of people with other sensory or physical impairments [20, 26, 56]. Our follow-up of some participants at 12 months (to be published separately) will provide preliminary evidence on the extent to which outcomes are maintained, continue to improve or deteriorate. Data gathered from our nested qualitative study will also be used to develop an initial theory of the ‘active ingredients’ of a hearing dog partnership, the processes by which changes in outcomes (or not) occur. This work will complement the trial findings and will usefully inform the design and scope of future studies.

It is important to note that hearing dog recipients are individuals who have actively pursued receiving a hearing dog. Thus findings cannot be applied to all those with this level of hearing loss. Indeed, it is likely that some of this population would not consider such an option due to personal preferences, living situation or cultural background [57]. In addition, some of this population have mobility or cognitive impairments that render them ineligible for such an intervention.

Our cost-effectiveness analysis implies that, given the current model of provision in the UK whereby the costs of hearing dogs are borne by the charitable sector (HDfDP), this intervention is a worthwhile investment when seen from a public sector perspective. Compared to usual care, hearing dogs reduced use and costs of public sector health and social care services, and improved QALY-based outcomes. Whilst it was not cost-effective for the full cost of hearing dogs to be borne by the health and social care sector, a partial contribution by the public sector of between £442 and £726 (dependent on health opportunity cost estimate), over the initial 6-month period of the hearing dog partnership could represent value for money (that is, health benefits would exceed the health opportunity costs). If the benefits and impacts on health observed in this study persisted over the duration of the hearing dog partnership (11 years on average), a contribution by the public sector of between £8,236 and £13,538 (calculated using an annual discount rate of 3.5%; figure dependent on measure of health opportunity cost and analysis considered) would represent value for money. As with other charities addressing health and social care needs, it would appear that the NHS and LAs benefit from the service provided by HDfDP. However, given its dependency on charitable donation, the extent to which HDfDP can exceed current rates of provision – and thus further benefit the public sector - is unclear.

Finally, we note that the trial evaluated the impacts of hearing dogs provided by Hearing Dogs for Deaf People. Assistance Dogs International (ADI) accredited hearing dog providers in other countries may differ with regard to training, assessment, matching and support processes. Findings may therefore not be applicable to hearing dogs provided by other ADI accredited organisations or other types of hearing dog providers.

## Limitations

Whilst the trial design and cost-effectiveness methods used are robust, there are a number of limitations. A hearing dog partnership typically lasts around 11 years. However, this trial only investigated the first six months into the partnership. (Pairs where the WL arm had not received their hearing dogs at twelve months were also followed up at this time point: these findings are reported elsewhere.) It is unlikely, however, that a trial design could be used to investigate longer-term outcomes.

Several HD arm participants did not receive a hearing dog within the data collection period. Consequently, we fell short of our target sample size (n = 128) at T1 meaning non-significant findings could be due to a lack of statistical power, rather than the absence of a true difference. The study only used self-report measures. Future studies could consider independent assessment of some outcomes (e.g. mental health, social interaction). The nature of the intervention, and the way it is provided, inherently introduce limitations that are not possible to remediate. Individuals in the WL arm had already been accepted for a hearing dog and had undergone HDfDP’s assessment process, thus they were not a true ‘no intervention’ comparator. However, we note that any impacts of this appear negligible (see Table 2). In addition, whilst study participants were blinded to which arm of the study they were in, it was not possible to blind them regarding whether they had received the intervention (a hearing dog) or not. Finally, this trial did not seek to compare receiving a hearing dog with pet dog ownership. Where study participants had previous experience of a pet dog, the qualitative element of this study (to be published separately) explored views on the differences between the two. Research, incorporating a comparative design, which sought to understand and measure the ‘added value’ of an assistance dog over a pet dog would be a useful addition to the growing evidence base on assistance dogs.

In taking a health and social care perspective to our cost-effectiveness evaluation, this study did not include the impact of hearing dogs on recipients’ productivity, nor the benefits and costs of informal care, nor the benefits and costs on family members or HDfDP volunteers involved in hearing dog training. If a broader perspective was adopted, these could be considered [58]. As a charity, HDfDP draws on charitable funding and volunteers’ input. If the budget and resource from charitable donors and volunteers were also considered, it becomes more complex to assess the opportunity cost (i.e. the benefits foregone) of investing in this intervention. To our knowledge, few full economic evaluations of charity-funded interventions have been undertaken and no method guidance has explicitly considered the opportunity cost implications arising from costs falling on charitable budgets.

To aid interpretation of cost-effectiveness results, we used an estimate of health opportunity costs (sometimes referred to as a cost-effectiveness threshold). This reflects how much benefit could be derived from alternative use of resources. At present, however, there is no empirical threshold value available for the health and social care sector combined. In the absence of this, we drew on widely used estimates of the health opportunity cost for the health care sector, which may not be representative of the social care sector. Finally, we note our estimates of the potential public sector contribution are subject to structural uncertainty because they assume effects on health observed during the trial period persist for the entire period of the hearing dog partnership. Whether this is the case is yet to be established.

## Conclusions

Findings indicate that, hearing dogs (provided by an organisation adhering to ADI standards) positively impact recipients’ mental well-being as well as daily functioning, mental health, experiences of fearfulness and social isolation and perceived dependency on others, at least in the short term. They suggest that hearing dogs are good value for money for the public sector but only if it does not bear the full cost of providing them. The study is an example of successful delivery of a trial in a third sector organisation with no previous experience of integrating a trial into its application, assessment and delivery processes. It also breaks new ground within the wider field of assistance dog evaluations.

## Supplementary Information

**Additional file 1.** CONSORT checklist.

**Additional file 2.** Health and social care unit costs.

**Additional file 3.** Cost-effectiveness analysis, supplementary tables (Health-related Quality of Life): Table 6: Health care resource use at each time point. Table 7: Social care resource use at each time point. Table 8: EQ-5D-5L index score, health and social care costs at each time point. Table 9: Missing data at each time point.

**Additional file 4.** Cost-effectiveness analysis, supplementary figure. (Health-related Quality of Life). Figure 2: Cost effectiveness plane and acceptability curve.

**Additional file 5.** Cost-effectiveness analysis, supplementary table (Social care related Quality of Life). Table 10: Cost-effectiveness analysis based on SC-QALYs: descriptive statistics and results.

**Additional file 6.** Cost-effectiveness analysis, supplementary figure (Health-related Quality of Life). Figure 3: Cost effectiveness plane and acceptability curve (Social Care-related Quality of Life).

## Data Availability

The datasets used and/or analysed during the current study are available from the corresponding author on reasonable request.

## Abbreviations

ADI: Assistance Dogs International
AUC: Area under the curve
BSL: British Sign Language
EQ-5D-5L: 5-level version of EuroQol 5 Dimensions instrument
GAD-7: Generalised Anxiety Disorder Assessment
HD: Hearing dog
HLQ: Hearing Loss Questionnaire
HRQoL: Health-related quality of life
PHQ-9: Patient Health Questionnaire-9
QALY: Quality-adjusted life year
RCT: Randomised controlled trial
SWEMWBS: Short Warwick-Edinburgh Mental Well-Being Scale
WL: Wait-list
WSAS: Work and Social Adjustments Scale
