# Correction to: Hearing dogs for people with severe and profound hearing loss: a wait-list design randomised controlled trial investigating their effectiveness and cost-effectiveness

**DOI:** 10.1186/s13063-021-05741-4

**Published:** 2022-01-25

**Authors:** Lucy Stuttard, Philip Boyle, Caroline Fairhurst, Catherine Hewitt, Francesco Longo, Simon Walker, Helen Weatherly, Emese Mayhew, Bryony Beresford

**Affiliations:** 1 Social Policy Research Unit, Department of Social Policy and Social Work, Church Lane Building, York Science Park, University of York, York, Heslington YO10 5DF UK; 2 York Trials Unit, University of York, York, YO10 5DD UK; 3 Centre for Health Economics, University of York, York, YO10 5DD UK

**Correction to: Trials 22, 700 (2021).**

**https://doi.org/10.1186/s13063-021-05607-9**

Following the publication of the original article [1], we were notified of the below:

1. The flow of participants from randomisation to T1 was missing in Fig. 1. Originally published vs. corrected Fig. 1 are presented below

**Fig. 1:**
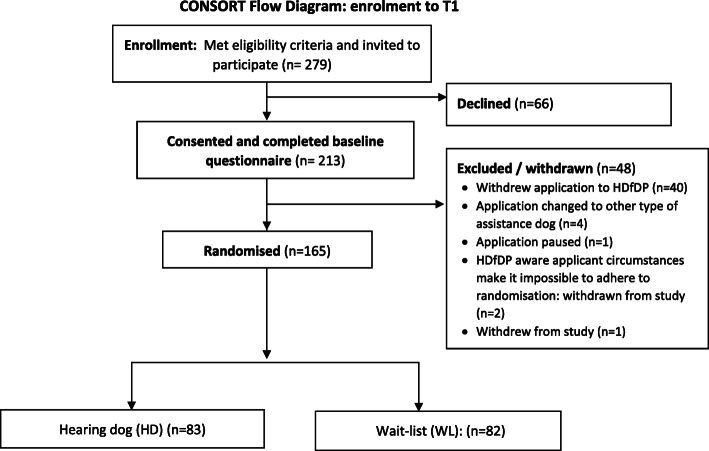
CONSORT Flow Diagram: enrolment to T1 (primary endpoint)

Originally published Fig. 1

Corrected Fig. 1

**Fig. 1:**
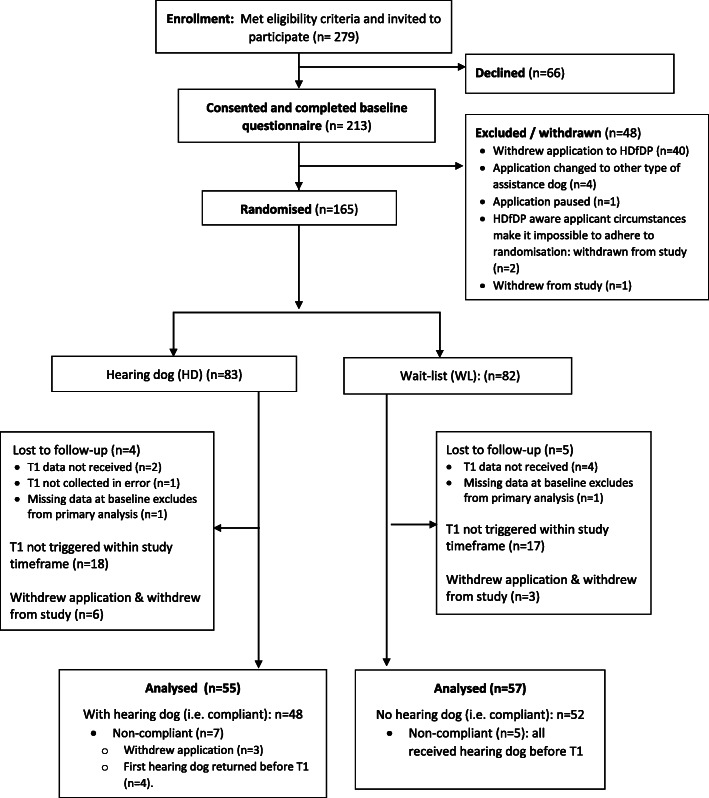
CONSORT Flow Diagram: enrolment to T1 (primary endpoint)

2. The formatting of Table 4 in the pdf needed adjustments.

3. Background: the final sentence of fourth paragraph:

“However, this did not include study participants (n = 3) with hearing dogs included in the effectiveness study [28].”

Should read:

“However, study participants (n = 3) with hearing dogs were not included in the effectiveness study [28].”

The original article has been corrected.

## References

[1] Stuttard et al. (2021) Hearing dogs for people with severe and profound hearing loss: a wait-list design randomised controlled trial investigating their effectiveness and cost-effectiveness (2021) 22:700 DOI: 10.1186/s13063-021-05607-9 10.1186/s13063-021-05607-9 PMC8515662 34649618

