# Supplementary material for: Hearing dogs for people with severe and profound hearing loss: a wait-list design randomised controlled trial investigating their effectiveness and cost-effectiveness
Source: Trials. 2021 Oct 14;22:700. doi: 10.1186/s13063-021-05607-9 (PMC8515662; doi:10.1186/s13063-021-05607-9)
Supplement: Supplementary file 2 — Additional file 2. Health and social care unit costs. [file 13063_2021_5607_MOESM2_ESM.docx]

**Additional file 2: Health and Social Care Unit Costs**

| Item | Unit cost (£) | Unit | Source | Notes |  |
| --- | --- | --- | --- | --- | --- |
| ***Primary or Community health care*** | | | | | |
| GP | 37.00 | Per visit | PSSRU 2018, p. 127 | General Practitioner. Cost per surgery consultation lasting 9.22 minutes including direct care staff costs and qualification costs. |  |
| Nurse (GP practice, district) | 10.85 | Per visit | PSSRU 2018, p. 125; PSSRU 2015, p. 174 | Nurse (GP practice), £42 of cost per hour including qualification costs; 15.5 minutes of contact duration (PSSRU, 2015). The calculations are £42*15.5/60. |  |
| ***Mental health and mental well-being services*** | | | | | |
| Mental health nurse | 44.67 | Per hour | PSSRU 2018, p. 123 | Community nurse. Average of Band 5, 6 and 7's cost per working hour. Qualification costs are included. |  |
| Counsellor | 43.67 | Per hour | PSSRU 2018, p. 119 | Scientific and professional staff. Average of Band 5, 6 and 7's cost per working hour. Qualification costs are included. |  |
| Psychologist | 53.00 | Per hour | PSSRU 2018, p. 119 | Scientific and professional staff. Band 7's cost per working hour. Qualification costs are included. |  |
| Psychiatrist | 109.00 | Per hour | PSSRU 2018, p. 161 | Hospital-based doctors. Psychiatric consultant's cost per working hour. Qualification costs are included. |  |
| Anti-depression medication prescription | 3.44 | Per month | <https://bnf.nice.org.uk/medicinal-forms/fluoxetine.html> | NHS indicative price for a one month prescription of Fluoxetine 20mg tablets, a widely prescribed anti-depressant (28 tablets). |  |
| Anti-anxiety medication prescription | 0.85 | Per month | [bnf.nice.org.uk/medicinal-forms/sertraline.html](https://bnf.nice.org.uk/medicinal-forms/sertraline.html) | NHS indicative price of a one month prescription of Sertraline (Zoloft) 100mg tablets, a widely prescribed anti-anxiety medication (28 tablets). |  |
| ***Outpatient appointments/services at hospital or specialist clinics*** | | | | | |
| Audiology service | 58.00 | Per visit | NHS Reference costs 2017/18, p. 8 | Average cost of community audiology care contact. |  |
| Specialist tinnitus service | 117.00 | Per visit | National Schedule of Reference Costs 2017/18, Total Other Currencies | Average cost of fitting of Hearing Aid or Device for Tinnitus (Currency code AS04). |  |
| Specialist cochlear implant centre | 327.99 | Per visit | National Schedule of Reference Costs 2017/18, Total Other Currencies | Average cost of maintenance and programming for a Cochlear Implant (Currency code AS13). |  |
| ENT clinic | 103.84 | Per visit | National Schedule of Reference Costs 2017/18, Total Outpatient Attendances | Activity-weighted average cost of consultant and non-consultant led ENT outpatient attendances (service code 120). |  |
| Any other outpatient attendances | 125.00 | Per visit | NHS Reference costs 2017/18,  p. 5 | Average cost of outpatient attendance. |  |
| ***Other hospital visits and hospital stays*** | | | | | |
| A&E attendance | 160.00 | Per visit | NHS Reference costs 2017/18,  p. 5 |  |  |
| Walk in centre (or similar) | 160.00 | Per visit | NHS Reference costs 2017/18,  p. 5 | Average cost of A&E attendance. |  |
| Day surgery/procedure | 742.00 | Per visit | NHS Reference costs 2017/18,  p. 5 |  |  |
| Inpatient hospital stays | 3,894.00 | Per visit | NHS Reference costs 2017/18,  p. 5 |  |  |
| Inpatient nights stayed in hospital | 431.00 | Per night | National Schedule of Reference Costs 2017/18, Index | Unit Cost of Elective Inpatients Excess Bed Days. |  |
| ***Council or local authority services*** | | | | | |
| Social worker | 84.00 | Per hour | PSSRU 2018, p. 139 | Social worker (adult services), cost per hour of client-related work including qualifications costs. |  |
| Occupational therapist | 47.00 | Per hour | PSSRU 2018, p. 142 | Community occupational therapist (local authority), cost per hour including training. |  |
| Equipment and assessment service (or similar) | 47.00 | Per hour | PSSRU 2018, p. 142 | Community occupational therapist (local authority), cost per hour including training. |  |
| Hearing loss team/service (or similar) | 47.00 | Per hour | PSSRU 2018, p. 142 | Community occupational therapist (local authority), cost per hour including training. |  |
| Drop in/advice service for deaf people/people with hearing loss | 47.00 | Per hour | PSSRU 2018, p. 142 | Community occupational therapist (local authority), cost per hour including training. |  |
| Home care service | 27.00 | Per hour | PSSRU 2018, p. 143 | Home care worker, cost of face-to-face per weekday hour. |  |
