# Supplementary material for: Hearing dogs for people with severe and profound hearing loss: a wait-list design randomised controlled trial investigating their effectiveness and cost-effectiveness
Source: Trials. 2021 Oct 14;22:700. doi: 10.1186/s13063-021-05607-9 (PMC8515662; doi:10.1186/s13063-021-05607-9)
Supplement: Supplementary file 3 — Additional file 3. Cost-effectiveness analysis, supplementary tables (Health-related Quality of Life): Table 6: Health care resource use at each time point. Table 7: Social care resource use at each time point. Table 8: EQ-5D-5L index score, health and social care costs at each time point. Table 9: Missing data at each time point. [file 13063_2021_5607_MOESM3_ESM.docx]

**Table 6: Health care resource use at each time point**

| Variable | Analysed sample | | | | | | HD arm | | | | |  | WL arm | | | | |
| --- | --- | --- | --- | --- | --- | --- | --- | --- | --- | --- | --- | --- | --- | --- | --- | --- | --- |
|  | All^a^ | |  | Users only^b^ | |  | All | |  | Users only | |  | All | |  | Users only | |
|  | Obs^c^ | Mean |  | Obs | Mean |  | Obs | Mean |  | Obs | Mean |  | Obs | Mean |  | Obs | Mean |
| *Baseline* | | | | | | | | | | | | | | | | | |
| Primary care |  |  |  |  |  |  |  |  |  |  |  |  |  |  |  |  |  |
| Appointments with GP | 156 | 1.9 |  | 104 | 2.8 |  | 79 | 1.8 |  | 55 | 2.5 |  | 77 | 2.0 |  | 49 | 3.2 |
| Appointments with nurse | 161 | 0.5 |  | 48 | 1.8 |  | 82 | 0.4 |  | 24 | 1.5 |  | 79 | 0.7 |  | 24 | 2.2 |
| Prescription for anti-depression medication | 163 | 0.3 |  | 49 | 1.0 |  | 83 | 0.3 |  | 27 | 1.0 |  | 80 | 0.3 |  | 22 | 1.0 |
| Prescription for anti-anxiety medication | 162 | 0.1 |  | 22 | 1.0 |  | 82 | 0.1 |  | 12 | 1.0 |  | 80 | 0.1 |  | 10 | 1.0 |
| Community care |  |  |  |  |  |  |  |  |  |  |  |  |  |  |  |  |  |
| Appointments with mental health nurse | 164 | 0.1 |  | 6 | 2.8 |  | 82 | 0.1 |  | 3 | 2.0 |  | 82 | 0.1 |  | 3 | 3.7 |
| Appointments with counsellor | 162 | 0.9 |  | 21 | 6.6 |  | 81 | 0.9 |  | 11 | 6.9 |  | 81 | 0.8 |  | 10 | 6.3 |
| Appointments with psychologist | 161 | 0.1 |  | 4 | 5.8 |  | 82 | 0.3 |  | 3 | 7.3 |  | 79 | 0.0 |  | 1 | 1.0 |
| Appointments with psychiatrist | 164 | 0.1 |  | 4 | 4.3 |  | 82 | 0.0 |  | 1 | 1.0 |  | 82 | 0.2 |  | 3 | 5.3 |
| Secondary care |  |  |  |  |  |  |  |  |  |  |  |  |  |  |  |  |  |
| Appointments with audiology service | 149 | 1.0 |  | 91 | 1.6 |  | 75 | 1.0 |  | 47 | 1.6 |  | 74 | 1.0 |  | 44 | 1.6 |
| Appointments with tinnitus service | 161 | 0.0 |  | 7 | 1.0 |  | 81 | 0.0 |  | 2 | 1.0 |  | 80 | 0.1 |  | 5 | 1.0 |
| Appointments with cochlear implant centre | 161 | 0.2 |  | 17 | 2.0 |  | 81 | 0.3 |  | 9 | 2.6 |  | 80 | 0.1 |  | 8 | 1.4 |
| Appointments with ENT clinic | 160 | 0.3 |  | 29 | 1.5 |  | 79 | 0.3 |  | 14 | 1.5 |  | 81 | 0.3 |  | 15 | 1.5 |
| Other outpatient appointments | 158 | 0.5 |  | 34 | 2.2 |  | 79 | 0.3 |  | 16 | 1.7 |  | 79 | 0.6 |  | 18 | 2.6 |
| A&E visits (no admission) | 163 | 0.2 |  | 18 | 1.5 |  | 82 | 0.1 |  | 9 | 1.2 |  | 81 | 0.2 |  | 9 | 1.8 |
| Walk in Centre (or equivalent) visits | 163 | 0.1 |  | 8 | 1.1 |  | 82 | 0.0 |  | 4 | 1.0 |  | 81 | 0.1 |  | 4 | 1.3 |
| Day case admissions | 164 | 0.1 |  | 10 | 1.9 |  | 82 | 0.1 |  | 5 | 1.2 |  | 82 | 0.2 |  | 5 | 2.6 |
| Approximate number of nights spent in hospital | 163 | 0.2 |  | 8 | 3.3 |  | 81 | 0.1 |  | 2 | 3.0 |  | 82 | 0.2 |  | 6 | 3.3 |
| *Randomisation* | | | | | | | | | | | | | | | | | |
| Primary care |  |  |  |  |  |  |  |  |  |  |  |  |  |  |  |  |  |
| Appointments with GP | 138 | 1.3 |  | 79 | 2.2 |  | 67 | 1.2 |  | 34 | 2.4 |  | 71 | 1.3 |  | 45 | 2.1 |
| Appointments with nurse | 139 | 0.5 |  | 33 | 2.2 |  | 68 | 0.5 |  | 14 | 2.6 |  | 71 | 0.5 |  | 19 | 1.9 |
| Prescription for anti-depression medication | 146 | 0.3 |  | 44 | 1.0 |  | 73 | 0.3 |  | 21 | 1.0 |  | 73 | 0.3 |  | 23 | 1.0 |
| Prescription for anti-anxiety medication | 146 | 0.1 |  | 16 | 1.0 |  | 73 | 0.1 |  | 8 | 1.0 |  | 73 | 0.1 |  | 8 | 1.0 |
| Community care |  |  |  |  |  |  |  |  |  |  |  |  |  |  |  |  |  |
| Appointments with mental health nurse | 146 | 0.0 |  | 3 | 1.3 |  | 73 | 0.0 |  | 0 | 0.0 |  | 73 | 0.1 |  | 3 | 1.3 |
| Appointments with counsellor | 143 | 0.5 |  | 12 | 6.5 |  | 72 | 0.6 |  | 6 | 7.5 |  | 71 | 0.5 |  | 6 | 5.5 |
| Appointments with psychologist | 144 | 0.0 |  | 1 | 1.0 |  | 72 | 0.0 |  | 0 | 0.0 |  | 72 | 0.0 |  | 1 | 1.0 |
| Appointments with psychiatrist | 146 | 0.0 |  | 5 | 1.2 |  | 73 | 0.0 |  | 2 | 1.5 |  | 73 | 0.0 |  | 3 | 1.0 |
| Secondary care |  |  |  |  |  |  |  |  |  |  |  |  |  |  |  |  |  |
| Appointments with audiology service | 138 | 0.7 |  | 61 | 1.5 |  | 68 | 0.7 |  | 34 | 1.5 |  | 70 | 0.6 |  | 27 | 1.6 |
| Appointments with tinnitus service | 141 | 0.0 |  | 4 | 1.0 |  | 70 | 0.0 |  | 2 | 1.0 |  | 71 | 0.0 |  | 2 | 1.0 |
| Appointments with cochlear implant centre | 139 | 0.2 |  | 16 | 1.4 |  | 69 | 0.2 |  | 8 | 1.5 |  | 70 | 0.2 |  | 8 | 1.4 |
| Appointments with ENT clinic | 140 | 0.2 |  | 18 | 1.4 |  | 69 | 0.2 |  | 9 | 1.4 |  | 71 | 0.2 |  | 9 | 1.3 |
| Other outpatient appointments | 134 | 0.5 |  | 25 | 2.5 |  | 65 | 0.3 |  | 10 | 2.2 |  | 69 | 0.6 |  | 15 | 2.7 |
| A&E visits (no admission) | 143 | 0.0 |  | 5 | 1.4 |  | 70 | 0.1 |  | 3 | 1.7 |  | 73 | 0.0 |  | 2 | 1.0 |
| Walk in Centre (or equivalent) visits | 144 | 0.1 |  | 8 | 1.6 |  | 71 | 0.1 |  | 4 | 1.8 |  | 73 | 0.1 |  | 4 | 1.5 |
| Day case admissions | 141 | 0.1 |  | 9 | 1.9 |  | 69 | 0.1 |  | 3 | 1.3 |  | 72 | 0.2 |  | 6 | 2.2 |
| Approximate number of nights spent in hospital | 144 | 0.2 |  | 8 | 3.0 |  | 71 | 0.0 |  | 1 | 2.0 |  | 73 | 0.3 |  | 7 | 3.1 |
| *T1* | | | | | | | | | | | | | | | | | |
| Primary care |  |  |  |  |  |  |  |  |  |  |  |  |  |  |  |  |  |
| Appointments with GP | 109 | 1.0 |  | 55 | 2.1 |  | 51 | 1.0 |  | 24 | 2.0 |  | 58 | 1.1 |  | 31 | 2.1 |
| Appointments with nurse | 105 | 0.4 |  | 27 | 1.7 |  | 50 | 0.4 |  | 12 | 1.5 |  | 55 | 0.5 |  | 15 | 1.9 |
| Prescription for anti-depression medication | 115 | 0.3 |  | 33 | 1.0 |  | 56 | 0.3 |  | 14 | 1.0 |  | 59 | 0.3 |  | 19 | 1.0 |
| Prescription for anti-anxiety medication | 115 | 0.2 |  | 18 | 1.0 |  | 56 | 0.1 |  | 7 | 1.0 |  | 59 | 0.2 |  | 11 | 1.0 |
| Community care |  |  |  |  |  |  |  |  |  |  |  |  |  |  |  |  |  |
| Appointments with mental health nurse | 113 | 0.2 |  | 4 | 5.0 |  | 55 | 0.3 |  | 2 | 7.5 |  | 58 | 0.1 |  | 2 | 2.5 |
| Appointments with counsellor | 112 | 0.6 |  | 12 | 5.9 |  | 55 | 0.8 |  | 6 | 7.0 |  | 57 | 0.5 |  | 6 | 4.8 |
| Appointments with psychologist | 113 | 0.1 |  | 3 | 2.0 |  | 56 | 0.0 |  | 0 | 0.0 |  | 57 | 0.1 |  | 3 | 2.0 |
| Appointments with psychiatrist | 113 | 0.2 |  | 4 | 4.5 |  | 55 | 0.1 |  | 1 | 3.0 |  | 58 | 0.3 |  | 3 | 5.0 |
| Secondary care |  |  |  |  |  |  |  |  |  |  |  |  |  |  |  |  |  |
| Appointments with audiology service | 109 | 0.7 |  | 47 | 1.6 |  | 52 | 0.7 |  | 23 | 1.5 |  | 57 | 0.7 |  | 24 | 1.7 |
| Appointments with tinnitus service | 112 | 0.0 |  | 1 | 1.0 |  | 54 | 0.0 |  | 1 | 1.0 |  | 58 | 0.0 |  | 0 | 0.0 |
| Appointments with cochlear implant centre | 110 | 0.1 |  | 10 | 1.3 |  | 53 | 0.1 |  | 6 | 1.0 |  | 57 | 0.1 |  | 4 | 1.8 |
| Appointments with ENT clinic | 112 | 0.2 |  | 10 | 1.8 |  | 54 | 0.1 |  | 4 | 1.8 |  | 58 | 0.2 |  | 6 | 1.8 |
| Other outpatient appointments | 105 | 0.5 |  | 23 | 2.3 |  | 49 | 0.2 |  | 4 | 2.3 |  | 56 | 0.8 |  | 19 | 2.3 |
| A&E visits (no admission) | 113 | 0.1 |  | 5 | 1.6 |  | 55 | 0.1 |  | 2 | 2.5 |  | 58 | 0.1 |  | 3 | 1.0 |
| Walk in Centre (or equivalent) visits | 112 | 0.0 |  | 4 | 1.3 |  | 55 | 0.0 |  | 2 | 1.0 |  | 57 | 0.1 |  | 2 | 1.5 |
| Day case admissions | 110 | 0.1 |  | 8 | 1.8 |  | 53 | 0.0 |  | 1 | 2.0 |  | 57 | 0.2 |  | 7 | 1.7 |
| Approximate number of nights spent in hospital | 112 | 0.0 |  | 0 | 0.0 |  | 55 | 0.0 |  | 0 | 0.0 |  | 57 | 0.0 |  | 0 | 0.0 |
| ^a^ All=total sample  ^b^ Users=study participants using/in receipt of service  ^c^ Obs=number of observations | | | | | | | | | | | | | | | | | |

**Table 7: Social care resource use at each time point**

| Variable | Analysed sample | | | | | | HD arm | | | | |  | WL arm | | | | |
| --- | --- | --- | --- | --- | --- | --- | --- | --- | --- | --- | --- | --- | --- | --- | --- | --- | --- |
|  | All^a^ | |  | Users^b^ only | |  | All | |  | Users only | |  | All | |  | Users only | |
|  | Obs | Mean |  | Obs | Mean |  | Obs | Mean |  | Obs | Mean |  | Obs | Mean |  | Obs | Mean |
| *Baseline* | | | | | | | | | | | | | | | | | |
| Appointments with social worker | 162 | 0.1 |  | 7 | 2.9 |  | 81 | 0.2 |  | 4 | 3.5 |  | 81 | 0.1 |  | 3 | 2.0 |
| Appointments with occupational therapist | 163 | 0.2 |  | 6 | 5.2 |  | 81 | 0.1 |  | 4 | 1.5 |  | 82 | 0.3 |  | 2 | 12.5 |
| Appointments with equipment and assessment service | 159 | 0.1 |  | 16 | 1.4 |  | 79 | 0.1 |  | 6 | 1.3 |  | 80 | 0.2 |  | 10 | 1.5 |
| Appointments with sensory impairment team (or equivalent) | 158 | 0.2 |  | 14 | 2.4 |  | 80 | 0.1 |  | 6 | 1.5 |  | 78 | 0.3 |  | 8 | 3.1 |
| Visits to LA drop in/advice service | 161 | 0.1 |  | 8 | 1.6 |  | 81 | 0.0 |  | 2 | 1.0 |  | 80 | 0.1 |  | 6 | 1.8 |
| Appointments with home care service | 162 | 1.4 |  | 3 | 73.0 |  | 80 | 1.6 |  | 2 | 64.5 |  | 82 | 1.1 |  | 1 | 90.0 |
| *Randomisation* | | | | | | | | | | | | | | | | | |
| Appointments with social worker | 144 | 0.2 |  | 2 | 15.5 |  | 72 | 0.4 |  | 1 | 30.0 |  | 72 | 0.0 |  | 1 | 1.0 |
| Appointments with occupational therapist | 145 | 0.1 |  | 5 | 2.2 |  | 72 | 0.1 |  | 4 | 2.5 |  | 73 | 0.0 |  | 1 | 1.0 |
| Appointments with equipment and assessment service | 142 | 0.1 |  | 10 | 1.1 |  | 72 | 0.1 |  | 7 | 1.1 |  | 70 | 0.0 |  | 3 | 1.0 |
| Appointments with sensory impairment team (or equivalent) | 143 | 0.1 |  | 11 | 1.5 |  | 72 | 0.2 |  | 6 | 1.8 |  | 71 | 0.1 |  | 5 | 1.0 |
| Visits to LA drop in/advice service | 143 | 0.1 |  | 11 | 1.4 |  | 72 | 0.1 |  | 5 | 1.4 |  | 71 | 0.1 |  | 6 | 1.3 |
| Appointments with home care service | 145 | 0.9 |  | 3 | 42.0 |  | 72 | 1.4 |  | 2 | 51.0 |  | 73 | 0.3 |  | 1 | 24.0 |
| *T1* | | | | | | | | | | | | | | | | | |
| Appointments with social worker | 114 | 0.0 |  | 2 | 1 |  | 56 | 0.0 |  | 1 | 1.0 |  | 58 | 0.0 |  | 1 | 1.0 |
| Appointments with occupational therapist | 113 | 0.0 |  | 1 | 1 |  | 54 | 0.0 |  | 0 | 0.0 |  | 59 | 0.0 |  | 1 | 1.0 |
| Appointments with equipment and assessment service | 112 | 0.1 |  | 5 | 1 |  | 56 | 0.1 |  | 2 | 1.5 |  | 56 | 0.1 |  | 3 | 1.0 |
| Appointments with sensory impairment team (or equivalent) | 111 | 0.1 |  | 7 | 2 |  | 55 | 0.0 |  | 1 | 1.0 |  | 56 | 0.2 |  | 6 | 1.8 |
| Visits to LA drop in/advice service | 114 | 0.2 |  | 6 | 4 |  | 56 | 0.3 |  | 2 | 7.5 |  | 58 | 0.1 |  | 4 | 2.0 |
| Appointments with home care service | 114 | 0.9 |  | 2 | 51 |  | 55 | 0.2 |  | 1 | 12.0 |  | 59 | 1.5 |  | 1 | 90.0 |
| a All=total sample  b Users=study participants using/in receipt of service  c Obs=number of observations | | | | | | | | | | | | | | | | | |

**Table 8: EQ-5D-5L index score, health and social care costs at each time point**

| Variable | Analysed sample | | | | |  | HD arm | |  | WL arm | |
| --- | --- | --- | --- | --- | --- | --- | --- | --- | --- | --- | --- |
|  | Obs^a^ | Mean | SD^b^ | Min | Max |  | Obs | Mean |  | Obs | Mean |
| *Baseline* | | | | | | | | | | | |
| EQ-5D score | 165 | 0.744 | 0.236 | -0.134 | 1.000 |  | 83 | 0.754 |  | 82 | 0.735 |
| Health care costs | 132 | 519 | 746 | 0 | 4,350 |  | 69 | 484 |  | 63 | 557 |
| Primary care costs | 154 | 75 | 90 | 0 | 751 |  | 78 | 71 |  | 76 | 78 |
| Community care costs | 160 | 55 | 144 | 0 | 763 |  | 81 | 60 |  | 79 | 50 |
| Secondary care costs | 141 | 379 | 685 | 0 | 4,350 |  | 72 | 353 |  | 69 | 406 |
| Social care costs | 149 | 83 | 335 | 0 | 2,729 |  | 73 | 80 |  | 76 | 85 |
| *Randomisation* | | | | | | | | | | | |
| EQ-5D score | 145 | 0.738 | 0.249 | -0.200 | 1.000 |  | 73 | 0.739 |  | 72 | 0.737 |
| Health care costs | 111 | 417 | 808 | 0 | 5,932 |  | 50 | 285 |  | 61 | 525 |
| Primary care costs | 133 | 53 | 72 | 0 | 555 |  | 64 | 50 |  | 69 | 55 |
| Community care costs | 143 | 29 | 100 | 0 | 568 |  | 72 | 32 |  | 71 | 25 |
| Secondary care costs | 121 | 354 | 786 | 0 | 5,705 |  | 56 | 207 |  | 65 | 481 |
| Social care costs | 139 | 61 | 306 | 0 | 2,567 |  | 72 | 97 |  | 67 | 23 |
| *T1* | | | | | | | | | | | |
| EQ-5D score | 114 | 0.741 | 0.235 | -0.218 | 1.000 |  | 56 | 0.785 |  | 58 | 0.698 |
| Health care costs | 83 | 363 | 507 | 0 | 2,658 |  | 42 | 231 |  | 41 | 499 |
| Primary care costs | 102 | 44 | 67 | 0 | 339 |  | 48 | 38 |  | 54 | 48 |
| Community care costs | 110 | 57 | 174 | 0 | 1,060 |  | 54 | 52 |  | 56 | 61 |
| Secondary care costs | 95 | 262 | 409 | 0 | 2,351 |  | 47 | 165 |  | 48 | 357 |
| Social care costs | 105 | 45 | 248 | 0 | 2,430 |  | 52 | 25 |  | 53 | 65 |
| ^a^ Obs=number of observations  ^b^ SD=standard deviation. | | | | | | | | | | | |

**Table 9: Missing data at each time point**

| Variable | Baseline | |  | At randomisation | |  | T1 | |
| --- | --- | --- | --- | --- | --- | --- | --- | --- |
|  | Obs^a^ | Prop^b^ |  | Obs | Prop |  | Obs | Prop |
| EQ-5D-5L utility-weighted score | 165 | 0.0% |  | 165 | 12.1% |  | 165 | 30.9% |
| Number appointments with GP | 165 | 5.5% |  | 165 | 16.4% |  | 165 | 33.9% |
| Number appointments with nurse | 165 | 2.4% |  | 165 | 15.8% |  | 165 | 36.4% |
| Received prescription for anti-depression medication | 165 | 1.2% |  | 165 | 11.5% |  | 165 | 30.3% |
| Received prescription for anti-anxiety medication | 165 | 1.8% |  | 165 | 11.5% |  | 165 | 30.3% |
| Cost of primary care | 165 | 6.7% |  | 165 | 19.4% |  | 165 | 38.2% |
| Number appointments with MH nurse | 165 | 0.6% |  | 165 | 11.5% |  | 165 | 31.5% |
| Number appointments with counsellor | 165 | 1.8% |  | 165 | 13.3% |  | 165 | 32.1% |
| Number appointments with psychologist | 165 | 2.4% |  | 165 | 12.7% |  | 165 | 31.5% |
| Number appointments with psychiatrist | 165 | 0.6% |  | 165 | 11.5% |  | 165 | 31.5% |
| Cost of community care | 165 | 3.0% |  | 165 | 13.3% |  | 165 | 33.3% |
| Number of appointments with audiology service | 165 | 9.7% |  | 165 | 16.4% |  | 165 | 33.9% |
| Number of appointments with tinnitus service | 165 | 2.4% |  | 165 | 14.5% |  | 165 | 32.1% |
| Number of appointments with cochlear implant centre | 165 | 2.4% |  | 165 | 15.8% |  | 165 | 33.3% |
| Number of appointments with ENT clinic | 165 | 3.0% |  | 165 | 15.2% |  | 165 | 32.1% |
| Number of other outpatient appointments | 165 | 4.2% |  | 165 | 18.8% |  | 165 | 36.4% |
| Number of A&E visits (not admitted) | 165 | 1.2% |  | 165 | 13.3% |  | 165 | 31.5% |
| Number of Walk in Centre (equivalent) visits | 165 | 1.2% |  | 165 | 12.7% |  | 165 | 32.1% |
| Number of times admitted as a day case | 165 | 0.6% |  | 165 | 14.5% |  | 165 | 33.3% |
| Approximate number of nights spent in hospital | 165 | 1.2% |  | 165 | 12.7% |  | 165 | 32.1% |
| Cost of secondary care | 165 | 14.5% |  | 165 | 26.7% |  | 165 | 42.4% |
| Cost of health care | 165 | 20.0% |  | 165 | 32.7% |  | 165 | 49.7% |
| Number of appointments with Social Worker | 165 | 1.8% |  | 165 | 12.7% |  | 165 | 30.9% |
| Number of appointments with Occupational Therapist | 165 | 1.2% |  | 165 | 12.1% |  | 165 | 31.5% |
| Number of appointments with Equipment and Assessment service | 165 | 3.6% |  | 165 | 13.9% |  | 165 | 32.1% |
| Number of appointments with Sensory Impairment Team | 165 | 4.2% |  | 165 | 13.3% |  | 165 | 32.7% |
| Number of visits to LA drop in/advice service | 165 | 2.4% |  | 165 | 13.3% |  | 165 | 30.9% |
| Number of appointments with Home care Service | 165 | 1.8% |  | 165 | 12.1% |  | 165 | 30.9% |
| Cost of social care | 165 | 9.7% |  | 165 | 15.8% |  | 165 | 36.4% |
| ^a^ Obs=number of users  ^b^ Prop=proportion of missing observations. | | | | | | | | |

Table 10

| Costing scenario^a^ | Trial Arm | | | | δ^QALY^ | | δ^COST^ | | ICER | Net Health Benefit (NHB) | | |
| --- | --- | --- | --- | --- | --- | --- | --- | --- | --- | --- | --- | --- |
|  | Hearing Dog | | Wait-list | |  |  |  |  |  | λ=£15,000 per QALY | λ=£20,000 per QALY | λ=£30,000 per QALY |
|  | QALYs | Costs | QALYs | Costs | Obs | Coeff | Obs | Coeff |  |  |  |  |
| *Multiple imputation analysis for the whole trial period* | | | | | | | | | | | | |
| Excluded | 1.395 | 3,909 | 1.347 | 4,407 | 165 | 0.011 | 165 | -260 | Dominant | 0.029 | 0.024 | 0.020 |
| Included |  | 7,123 |  |  |  |  |  | 2,954*** | 262,375 | -0.186 | -0.136 | -0.087 |
| *Multiple imputation analysis for the active intervention period* | | | | | | | | | | | | |
| Excluded | 0.399 | 911 | 0.380 | 1,206 | 165 | 0.013 | 165 | -291 | Dominant | 0.032 | 0.027 | 0.022 |
| Included |  | 4,125 |  |  |  |  |  | 2,954*** | 232,112 | -0.184 | -0.135 | -0.086 |
| *Key:* |  |  |  |  |  |  |  |  |  |  |  |  |
| δ=adjusted mean difference | | | Obs=number of observations used to estimate δ | | | | | | ICER=incremental cost-effectiveness ratio | | | |
| λ=opportunity-costs threshold | | | Coeff=estimated coefficient δ | | | | | |  |  |  |  |
| *** p-value=0.01 | | | | | | | | | | | | |
