# Supplementary figures and images for: Hearing dogs for people with severe and profound hearing loss: a wait-list design randomised controlled trial investigating their effectiveness and cost-effectiveness
Source: Trials. 2021 Oct 14;22:700. doi: 10.1186/s13063-021-05607-9 (PMC8515662; doi:10.1186/s13063-021-05607-9)

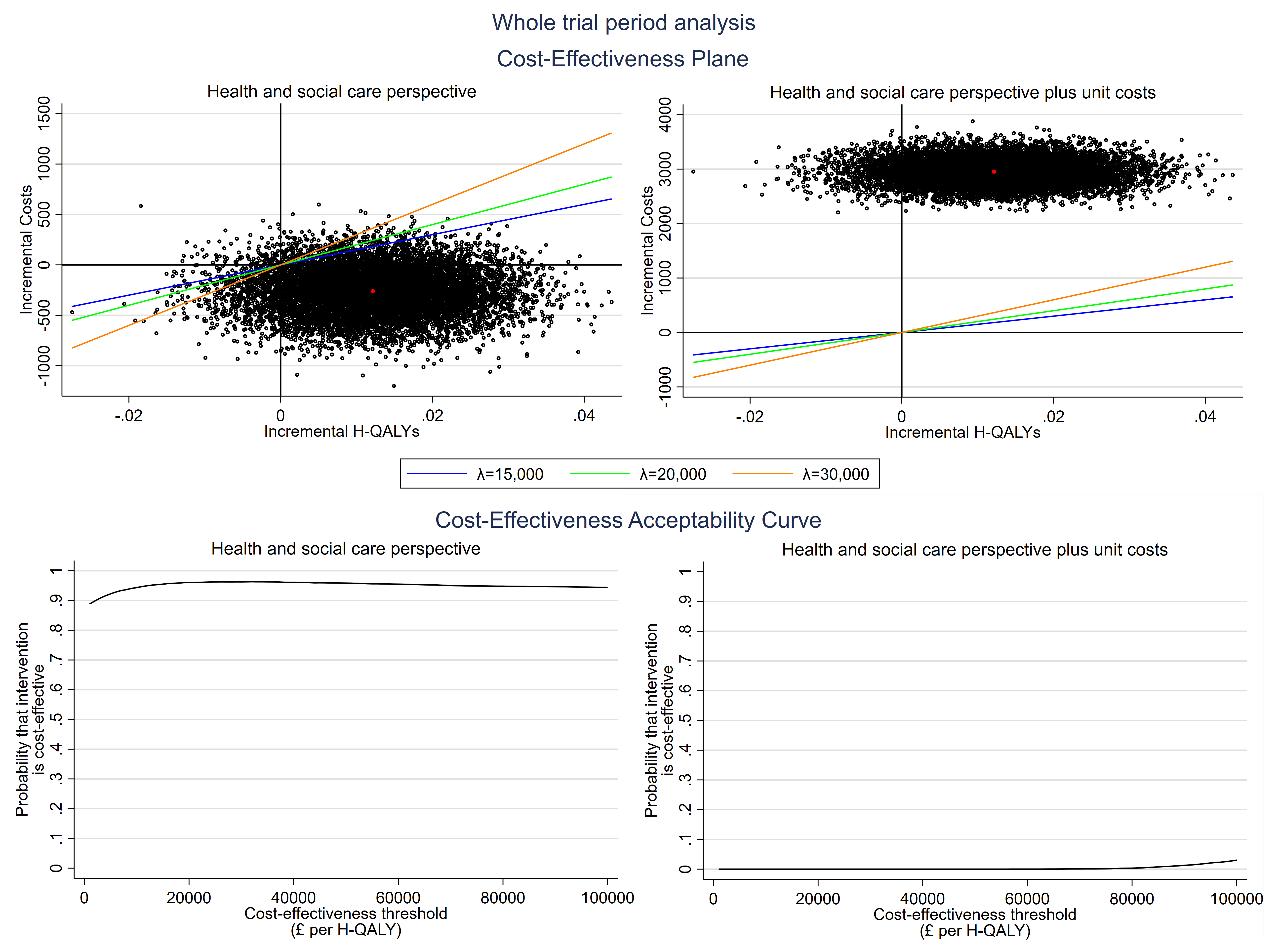

Supplement: Supplementary file 4 — Additional file 4. Cost-effectiveness analysis, supplementary figure. (Health-related Quality of Life). Figure 2: Cost effectiveness plane and acceptability curve. [file 13063_2021_5607_MOESM4_ESM.png]

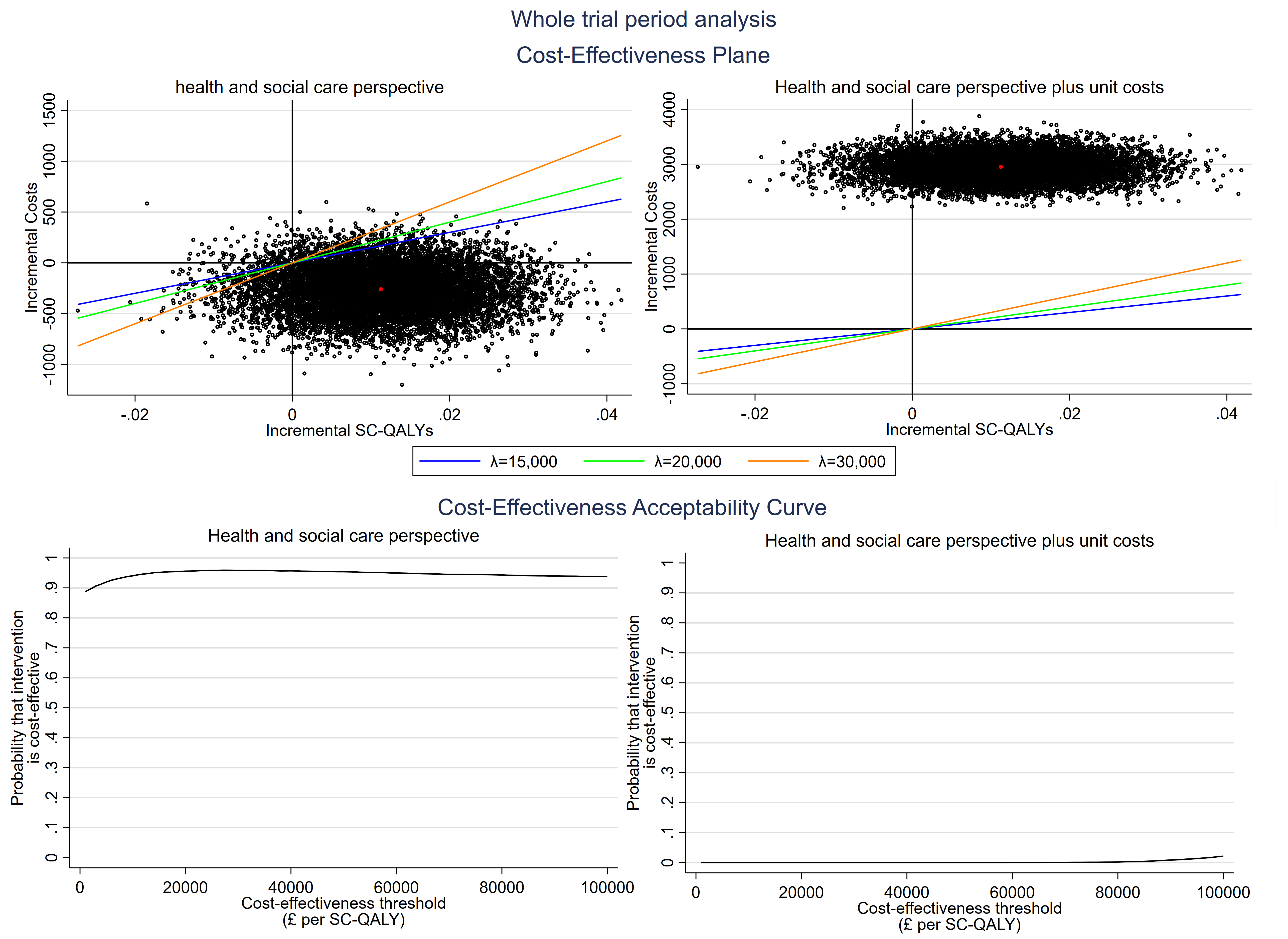

Supplement: Supplementary file 6 — Additional file 6. Cost-effectiveness analysis, supplementary figure (Health-related Quality of Life). Figure 3: Cost effectiveness plane and acceptability curve (Social Care-related Quality of Life). [file 13063_2021_5607_MOESM6_ESM.png]
