# Supplementary material for: Hearing dogs for people with severe and profound hearing loss: a wait-list design randomised controlled trial investigating their effectiveness and cost-effectiveness
Source: Trials. 2021 Oct 14;22:700. doi: 10.1186/s13063-021-05607-9 (PMC8515662; doi:10.1186/s13063-021-05607-9)
Supplement: Supplementary file 5 — Additional file 5. Cost-effectiveness analysis, supplementary table (Social care related Quality of Life). Table 10: Cost-effectiveness analysis based on SC-QALYs: descriptive statistics and results. [file 13063_2021_5607_MOESM5_ESM.docx]

**Table 10: Cost-effectiveness analysis based on SC-QALYs: descriptive statistics and results**

| Costing scenario^a^ | Trial Arm | | | | δ^QALY^ | | δ^COST^ | | ICER | Net Health Benefit (NHB) | | |
| --- | --- | --- | --- | --- | --- | --- | --- | --- | --- | --- | --- | --- |
|  | Hearing Dog | | Wait-list | |  |  |  |  |  | λ=£15,000 per  SC-QALY | λ=£20,000 per  SC-QALY | λ=£30,000 per  SC-QALY |
|  | SC-QALYs | Costs (£) | SC-QALYs | Costs (£) | Obs | Coeff | Obs | Coeff |  |  |  |  |
| *Multiple imputation analysis for the whole trial period* | | | | | | | | | | | | |
| Excluded | 1.395 | 3,909 | 1.347 | 4,407 | 165 | 0.011 | 165 | -260 | Dominant | 0.029 | 0.024 | 0.020 |
| Included |  | 7,123 |  |  |  |  |  | 2,954*** | 262,375 | -0.186 | -0.136 | -0.087 |
| *Multiple imputation analysis for the active intervention period* | | | | | | | | | | | | |
| Excluded | 0.399 | 911 | 0.380 | 1,206 | 165 | 0.013 | 165 | -291 | Dominant | 0.032 | 0.027 | 0.022 |
| Included |  | 4,125 |  |  |  |  |  | 2,954*** | 232,112 | -0.184 | -0.135 | -0.086 |
| *Key:* |  |  |  |  |  |  |  |  |  |  |  |  |
| δ=adjusted mean difference | | | Obs=number of observations used to estimate δ | | | | | | ICER=incremental cost-effectiveness ratio | | | |
| λ=opportunity-costs threshold | | | Coeff=estimated coefficient δ | | | | | |  |  |  |  |
| *** p-value=0.01 | | | | | | | | | | | | |

^a^ Refers to two scenarios used for each analysis: costs of providing a hearing dog excluded (i.e. borne by HDfDP charity: the current situation),

or included in costs to public sector (health and social care).
